# ESCAlate – Adaptive treatment approach for adolescents and adults with ADHD: study protocol for a randomized controlled trial

**DOI:** 10.1186/s13063-018-2665-9

**Published:** 2018-05-18

**Authors:** Toivo Zinnow, Tobias Banaschewski, Andreas J. Fallgatter, Carolin Jenkner, Florence Philipp-Wiegmann, Alexandra Philipsen, Wolfgang Retz, Esther Sobanski, Johannes Thome, Michael Rösler

**Affiliations:** 1 Institute for Forensic Psychology and Psychiatry, Saarland University, Kirrberger Straße, 66421 Homburg, Saarland Germany; 2 Department of Child and Adolescent Psychiatry and Psychotherapy, Central Institute of Mental Health, Medical Faculty Mannheim, Heidelberg University, J5, 68159 Mannheim, Germany; 3 Department of Psychiatry, University of Tübingen, Osianderstr. 24, 72076 Tübingen, Germany; 4 LEAD Graduate School, University of Tübingen, Tübingen, Germany; 5 Clinical Trials Unit at University Medical Center Freiburg, Elsässer Str. 2, 79110 Freiburg, Germany; 6 Department of Psychiatry and Psychotherapy, University of Bonn, Siegmund-Freud-Straße 25, 53127 Bonn, Germany; 7 Department of Psychiatry and Psychotherapy, University Medical Center Mainz, Untere Zahlbacherstraße 8, 55131 Mainz, Germany; 8 Department of Psychiatry and Psychotherapy, Central Institute of Mental Health, Medical Faculty Mannheim, Heidelberg University, J5, 68159 Mannheim, Germany; 9 Department of Child and Adolescent Psychiatry and Psychotherapy, University Medical Center Mainz, Mainz, Germany; 10 Department of Psychiatry and Psychotherapy, University Hospital Rostock, Gehlsheimer Straße 20, 18147 Rostock, Germany

**Keywords:** ADHD, Attention deficit hyperactivity disorder, Adolescence, Adult, Psychoeducation, Neurofeedback, Pharmacological treatment, Randomized controlled trail, RCT

## Abstract

**Background:**

Over the last decade, a wide range of attention-deficit/hyperactivity disorder (ADHD) treatment approaches for adults, including both pharmacological interventions and psychosocial treatments, have been proposed and observed to be efficient. In practice, individual treatment concepts are based on results of clinical studies as well as international guidelines (NICE Guidelines) that recommend a step-by-step treatment approach. Since the evidence supporting this approach is limited, the aim of the present study is to determine an optimal intervention regarding severity levels of ADHD symptomatology conducting a randomized controlled trial.

**Method:**

We aim to include 279 ADHD subjects aged between 16 and 45 years. First, participants are randomized to either a face-to-face psychoeducation, telephone assisted self-help (TASH), or a waiting control group (Step 1). All participants assigned to the control group are treated using TASH after a 3-month waiting period. Participants are then allocated to one of three groups, based on their remaining severity level of ADHD symptoms, as (1) full responder, (2) partial responder, or (3) non-responder (Step 2). Full responders receive counseling, partial responders receive either counseling only or counseling and neurofeedback (NF), and non-responders receive either pharmacological treatment only or pharmacological treatment and NF, followed by a 3 month period without intervention.

**Discussion:**

The naturalistic sample is one of the study’s advantages, avoiding highly selective inclusion or exclusion criteria. The efficacy of an evidence-based stepped care intervention is explored by primary (reduction of severity of ADHD symptoms) and secondary outcomes (functional outcomes, e.g., quality of life, anger management, enhancement of psychosocial well-being). Predictors of therapeutic response and non-response are being investigated at each step of intervention. Further, sex differences are also being explored.

**Trial registration:**

This study is registered by the German Trial Register (reference number: DRKS00008975), 23 October 2015.

**Electronic supplementary material:**

The online version of this article (10.1186/s13063-018-2665-9) contains supplementary material, which is available to authorized users.

## Background

With up to 5.3% of children affected worldwide, attention-deficit/hyperactivity disorder (ADHD) is one of the most common diseases with early childhood onset [1]. ADHD has long been considered a disease of childhood, with full remission in adulthood, with adult ADHD still being questioned by many practitioners. However, clinical long-term studies have shown that symptoms fully, or at least partially, persist into adulthood in 60% of ADHD patients [2]. Cross-section analyses support this data. While approximately 3% to 7% of school-aged children are affected by ADHD [3], prevalence in young and middle adulthood, as well as in seniority, ranges between 2% and 5% [1, 4, 5].

### Pharmacological treatment

Several meta-analyses provided evidence that short to medium term pharmacological interventions, using stimulant medication or atomoxetine as well as psychotherapeutic treatment strategies, will improve psychopathological symptoms of adult ADHD [6, 7]. Recent investigations have revealed smaller effects in psychosocial interventions than in pharmacological treatment [6, 7]. However, effect sizes attained by pharmacological treatments have been found to be smaller for adults than for adolescents. Additionally, results of pharmacological trials are generally influenced by study sample selectivity and pre-defined study approaches, following strict inclusion and exclusion criteria such as comorbidities. Therefore, the characteristics of patients to be included in the current study will not necessarily match those of an average patient with ADHD.

### Psychosocial treatment

Regarding low level psychosocial treatment options, psychoeducation (PE) is well established in daily practice [8]. Additionally, telephone assisted self-help (TASH) interventions have been found to be useful for children and adolescents [9], while they have only been used for patient parent education. This is a different approach than in adult psychiatry, where ADHD patients receive treatment directly. However, knowledge about the efficacy of TASH in adults is limited and promising results attained by childhood studies cannot yet be transferred to adult patients. To date, only one randomized controlled pilot study has been published, evaluating an 8-week bibliotherapy program for adults, in which therapist contact was minimized to phone call sessions. This study, conducted by Stevenson et al. [10], provides first promising results suggesting significant improvements of ADHD symptoms through TASH and the concept displays high similarities with the program introduced herein [8].

### Neurofeedback (NF) training

Regarding NF training, impaired regulations of slow cortical potentials (SCPs), as well as reduced negativities in anticipation of tasks, have been observed through electroencephalogram (EEG) recordings for adults and adolescents as compared to children with ADHD. The most consistent neurophysiological finding in children with ADHD is an increased absolute power in the theta EEG band [11–20]; further, several studies have also found decreased activity in the beta band [19–21]. For adult ADHD patients, increased absolute power in the alpha and theta EEG were observed compared to control groups, while there were no differences in the beta activity [22, 23]. Thus, the EEG of adult ADHD patients is characterized by an increased share of slow potentials and does not show the same abnormality in beta activity as in children with ADHD [11, 22]. This normalization of beta activity is associated with a reduced hyperactivity in adults with ADHD [11, 22].

Strehl et al. [24] investigated the effects of self-regulation of SCP for children with ADHD. Measurement before and after the trials showed that children with ADHD are able to learn to regulate negative SCPs. After training, significant improvement in behavior, attention, and IQ score were observed.

Based on these results, NF trainings were developed to augment the cortical activation necessary for focused attention and cognitive tasks. In previous research, the use of SCP as a treatment parameter in NF applications has been associated with a significant reduction of ADHD symptoms such as improved attention variables [25] and corresponding event-related potential (ERP) changes. EEG recordings during SCP treatment indicated that children with ADHD were able to control their SCPs after training, a skill that remained stable after a 2-year follow-up period. Studies assessing SCP training in adult patients with ADHD are scarce, but preliminary data based on 10 adult patients, showed that 15 sessions of SCP training led to significant improvements regarding self-ratings of ADHD symptoms. A trend towards normalization of contingent negative variation amplitudes (CNV; an event-related potential (ERP) related to aspects of action preparation and self-regulation) could also be observed [26]. Meta-analytic results, attained by Sonuga-Barke et al. [27] revealed medium to large effects (d = 0.59) for children with ADHD, while the majority of randomized controlled studies (RCTs) included medicated patients. The authors also report that studies conducted in a (probably) blinded manner yielded smaller effects (d = 0.29), reaching only a statistical trend. Further, they indicated that this estimate may be too low due to less sensitive ratings and non-standardized NF [28]. The largest study employing standard NF and sensitive ratings, as proposed herein, reports considerably larger effects (parent rating: d = 0.64, [25]; teacher rating: d = 0.42, as calculated in [27]). Effect sizes shown for NF plus medication as compared to medication alone vary widely, ranging between small (d = 0.46, [29]) and large effects (d = 2.2, [30, 31]). Meta-analytic estimates could not be obtained in this case because many NF RCTs did not exclude patients with concurrent medication [27]. In conclusion, there is a wide range of incremental NF effects that cover potentially large and clinically highly relevant effects. Until now, only few studies have focused on this topic, while none of them have included adult samples. Therefore, the current study aims to investigate potential effects of NF, along with individual response predictors, in a stepped care design.

### Combined therapy

There is ample evidence surrounding the effects of respective treatment options, yet the number of studies regarding combinations of psychosocial intervention and pharmacological treatment or even the stepped care model, as suggested here, is very limited. Concerning the combination of medication and psychosocial treatment in children and adolescents, the MTA study [32] demonstrated a superiority of stimulant medication over psychosocial interventions, wherein patients responding to stimulant treatment did not experience additional benefit when treated with behavior therapy. A recent study of our consortium compared a structured group program therapy based on cognitive behavioral therapy (CBT) and dialectic behavior therapy with individual clinical management in combination with methylphenidate (MPH) or placebo in a factorial four condition design [33]. This study has been granted by the BMBF (*Bundesministerium für Bildung und Forschung*, Registration: CCT-ISRCTN54096201). Results suggest that group program therapy is not superior compared to clinical management regarding the primary outcome (ADHD symptoms after 3 months of intense treatment), whereas MPH is superior to placebo [34]. Two studies conducted by Safren et al. [35] and Emilsson et al. [36] employed a different design, comparing patients treated with ADHD medication (mostly stimulants) only and patients receiving medication as well as individual [35] or group [36] CBT. Both research groups were able to attain a significantly more pronounced reduction of ADHD psychopathology for patients through combined treatment.

### Study objectives

The limited data regarding combined treatments and the implementation of a combination of pharmacological and non-pharmacological treatment options in a stepped care model are the point of origin of our study concept, which holds promise for improving the knowledge on individual treatment optimization for adult ADHD patients in outpatient facilities. Further, prevailing research does not yield conclusive results regarding the prediction of response or non-response to psychosocial or pharmacological treatment. The role of psychosocial and biological predictors such as age, sex, compliance, comorbidities, lifestyle, adverse environment, or psychosocial state is yet to be resolved. The majority of studies concerning predicting factors have used data from pharmacological trials in an ex-post design to identify said factors, leading to the assumption that those studies had primarily been designed to demonstrate pharmacological efficacy. Since the question of possible differences concerning therapeutic needs between male and female patients is almost unmet in relevant research, sex effects will be of particular interest in the current study. Thus, we expect to attain new information regarding sex effects and other confounding variables on treatment adherence and outcome in a sample consisting of adult ADHD patients.

**Fig. 1:**
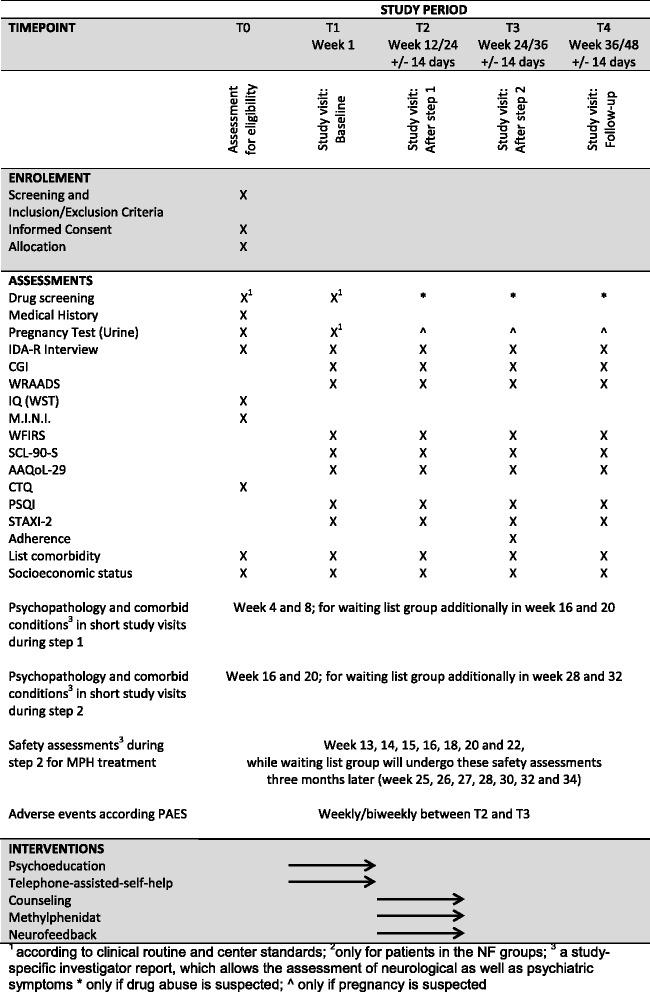
Spirit 2013 flow diagram

The paper reports the ESCAlate trial protocol (**E**vidence-Based, **S**tepped-**C**are in **Late** Adolescents and Young **A**dults with Attention-Deficit/Hyperactivity-Disorder, Version 05, from December 20, 2016) and has been conceived under consideration of the SPIRIT guidelines [37, 38] (Fig. 1 and Additional file 1).

### ESCAlife

ESCAlate is part of the research consortium ESCAlife, whose primary aim is to investigate adaptive interventions for patients with ADHD from preschool age to adulthood. Besides ESCAlate, ESCAlife encompasses ESCApreschool for children aged between 3 and 5 years, ESCAschool for children aged between 6 and 12 years, and ESCAadol for adolescents aged between 12 and 15 years. Trial protocols of those accompanying studies are reported elsewhere.

## Method

### Study conduct and trial flow

The present study is a multisite trial, gathering evidence via six different recruiting centers adjoined to university hospitals across Germany (Homburg/Saar, Mainz, Mannheim, Oldenburg, Rostock, Tübingen). The coordinating center is the Institute for Forensic Psychology and Psychiatry, University of Saarland. To reach a sample size of *N* = 279 patients with ADHD, as diagnosed according to DSM-5 (baseline; T0), we will be able to mobilize further recruitment centers if needed. Inclusion criteria are patient age (16–45 years), their or a guardian’s informed consent, and diagnosable ADHD, assessed through a structured clinical interview (Integrated Diagnostic Scale of adult ADHD – Revised; IDA-R) [39–41]. Exclusion criteria consist of IQ < 80 (according to WST [42]), psychiatric disorders (e.g., schizophrenia, acute psychotic disorders, mania, bipolar disorder, affective disorders, antisocial personality disorder), and medication (psychotropic or ADHD). Patients using psychotropic or ADHD medication need to undergo a 4-week wash-out period before they are eligible for participation in the study. Further exclusion criteria are current alcohol or drug dependence, severe heart disease, epilepsy, insufficient command of the German language, pregnancy, or breast feeding. Common comorbid conditions such as conduct disorder, personality disorders (except antisocial personality disorder), anxiety disorder or mild to moderate depression as well as substance use disorder in remission do not lead to exclusion.

In a first step (T1), patients will be equally randomized to the two active treatments or waiting control groups. One-third of patients will be assigned to face-to-face PE, which is being implemented through standardized modules [8]. Eight individual sessions will be administered with each patient over a period of 3 months. The competing treatment is TASH, which essentially consists of the same standardized modules as the face-to-face PE, yet implemented through 30 min telephone sessions. Those modules include a structured procedure, defining primary problems that provide specific information concerning ADHD, present coping skills based on CBT, and require written homework. A third group of patients will be wait-listed; serving as a control group, these patients will not receive treatment for 3 months, and will subsequently receive TASH. Thus, the duration of step 1 is 3 months for PE and TASH groups and 6 months for the waiting list control group. We hypothesize that both PE and TASH will display superior effects in terms of ADHD symptom reduction as compared to wait-listed patients after treatment.

Using the IDA-R, treatment response will then be determined (T2). Patients fulfilling our a priori criterion of full response (IDA-R score ≤ 18) will be followed up by monthly clinical routine sessions in which they get interviewed by a clinical doctor to control for psychopathological status and to prevent relapse. During this follow-up period, no ADHD medication or psychotherapeutic interventions are allowed. However, in case of relapse or the emergence of comorbid conditions, all treatment options fit to improve the patient’s situation are permitted.

Patients categorized as partial responders (IDA-R score 19–27) will be randomly allocated to either individual counseling or individual counseling in combination with NF, with each intervention being conducted over a period of 3 months (step 2). As for NF training, following the standard protocol as proposed by Mayer et al. [26], SCPs will be recorded at Cz (vertex region), then referenced against mastoid A1 with a ground electrode on mastoid A2, and averaged. This will be performed using NEURO PRAX^®^ (neuroConn group), a full-band DC-EEG system, primarily intended for neuroscientific application. For all channels, physiological signals, such as EEG, electromyogram (EMG), and evoked potentials (EP), are measured simultaneously and synchronously, in the frequency range of 0 to 1200 Hz. EEG activities, from the infra-slow (0 to 0.3 Hz) to the ultra-fast (80 to 1200 Hz), are captured by the unique amplifier technology. Various software and hardware modules are able to make online correction of artifacts caused by muscle and eye movements, conduct topographical analyses or spectral and amplitude mapping, as well as online averaging and bio-feedback and NF. Each training session is composed of four runs of 40 trials, with each trial lasting 8 seconds and consisting of three stages, namely a baseline phase (0–2 s), an active phase (2–7.5 s), and a reinforcement phase (7.5–8 s). At the end of the baseline period, participants will be presented with a cue, namely a triangle pointed upwards indicating ‘activation’ (regulate a negative SCP shift) or pointed downwards indicating ‘deactivation’ (regulate a positive SCP shift). In this active phase, an object moving up or down across the screen will indicate activation or deactivation, thus providing feedback to the participant. In a third phase, participants will be positively reinforced receiving visual rewards in 75% of cases in which they successfully directed their SCP activity. In 25% of trials (‘transfer trials’) there will be no feedback, ensuring that the application of regulation does not depend on the reward system but on acquired skills. The NF training employed herein comprises 25 sessions in 3 months, with sessions 1–12 being composed of activation and deactivation trials equally. In sessions 13–25, the ratio changes to 40% and 60%, respectively. Training sessions will take place once or twice a week and last approximately 1 hour each, including preparation time. Using a waiting list control group or another passive control group in the second treatment step can be problematic, as patients not responding sufficiently to therapy in step 1 would be without substantial treatment for 3 months, despite an urgent need for treatment. In order to avoid this, an individual counseling program was introduced to the study plan. Patients randomly assigned to the counseling program or counseling and NF will receive six individual sessions of 30 min, allowing them to address ADHD-related symptoms as well as individual, everyday functional problems. The intervention program for counseling in a one-to-one setting is manual-based but not yet published. It is based on the manual for group therapy with adult patients with ADHD by D’Amelio et al. [8]. We hypothesize that NF in combination with counseling is superior to counseling alone, in reducing ADHD psychopathology.

**Fig. 2:**
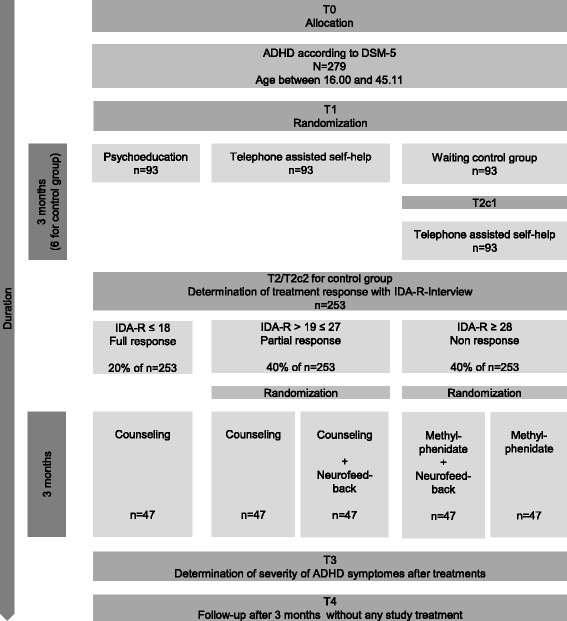
Steps of the ESCAlate treatment program

If patients do not respond to treatment obtained in step 1, they will be considered non-responders (IDA-R score ≥ 28) and will then be equally randomized to either a group treated with MPH only or a group treated with MPH in combination with NF training. MPH has been chosen as medical treatment because, according to evidence-based treatment guidelines (NICE Guidelines) [43], MPH is first line when it comes to ADHD medication. Medication administration will be coordinated by each patient’s attending physician as part of their routine care as recommended by current treatment guidelines and according to the product information sheet, as well as the dosage schedule defined by the BfArM (Bundesinstitut für Arzneimittel und Medizinprodukte). Specifically, the patients’ medical treatment will begin with a dosage of 10 mg MPH in the morning and will be increased in steps of 10 mg, with the highest possible dosage of 80 mg per day. If significant side effects are reported, dosage will be again decreased in steps of 10 mg until an appropriate level of medication is found. The optimal daily dosage will then be maintained and the number of subdoses taken during the day will be decided individually by the respective medical doctor. A step-by-step overview of the study process is shown in Fig. 2. Trial time flow is illustrated in Table 1.

**Table 1:** ESCAlate timeline flow

	1st year				2nd year				3rd year				4th year			
Months	1	4	7	10	13	16	19	22	25	28	31	34	37	40	43	47
Preparation (incl. adaptation of treatment manuals, therapist training, study protocol, electronic case report form)	x	x														
Initiation of sites			x													
Recruitment				x	x	x	x	x	x	x	x					
Clinical conduct (treatment and follow-up period)				x	x	x	x	x	x	x	x	x	x	x	x	
Database clearing																x
Data analysis, publication																x

### Assessment scope

During step 1, patients will be assessed regarding their ADHD psychopathology and comorbid conditions at baseline and in treatment weeks 4, 8, and 12. Patients allocated to the waiting list group will additionally be evaluated at weeks 16, 20, and 24. After the T2 assessment at the end of step 1, patients who are subsequently being treated with MPH or a combination of MPH and NF will be reassessed at T3 regarding their ADHD psychopathology, comorbid conditions, adverse events, and adherence. Patients treated pharmacologically will undergo safety assessments during step 2 in weeks 13, 14, 15, 16, 18, 20, 22, and 24, while those in the waiting list control group will undergo each procedure 3 months later. An identical schedule is being implemented for patients receiving NF training or NF in combination with individual counseling.

### Psychometric measures and psychophysiological data

#### Primary outcome

The IDA-R [39–41] is a structured interview assessing ADHD symptomology and allowing for subtype diagnosis following DSM-5 criteria. Essentially, this tool is an interview version of the ADHD-Diagnosis Checklist (ADHD-DC) [44], which has been successfully used in various multicenter studies concerning ADHD (e.g., EMMA [45]). Compared to the ADHD-DC, IDA-R comes with a higher user applicability, as the questions are already formulated. The examiner estimates the manifestation of the ADHD pathology based on 18 items. These items are rated on a scale from ‘0’ to ‘3’, while with a score of ‘2’ the diagnostic criteria of the DSM-5 is already met (for the specific item). Nine items are assigned to the inattentive and nine items to the hyperactive-impulsive type of ADHD. An ADHD diagnosis can be made when at least five items are rated with a score of ‘2’ in one of the two subscales. While there is no simple cut-off score for diagnosis, the sum score is only used to differentiate the severity level after the diagnosis has already been made. The quality criteria, objectivity, reliability, and validity of the IDA-R, based on the ones of ADHS-DC, are met.

#### Secondary outcomes

The Clinical Global Impression Scale (CGI) [46] is a single question, observer rating scale evaluating the severity of illness on a 7-point scale. Conducted by clinicians who are blind to treatment condition, patients are being assessed judging functional impairment, symptom severity, and distress or coping. The CGI has been used in several treatment evaluation studies and has been found to correlate with ADHD severity measures (e.g., adult ADHD investigator symptom rating scale [47]).

The Weiss Functional Impairment Rating Scale (WFIRS-S) [48] is a self-rating scale appraising functional impairment in seven domains of everyday life (family, work, school, life skills, self-concept, social, risk). As part of the recommended diagnostic rating scales provided by the Canadian Evidence-based and Expert Guidelines, the WFRIS-S is a widely accepted instrument.

The Wender Reimherr Adult Attention Deficit Disorder Scale (WRAADS) [49] is a structured interview assessing attention deficits, such as motor hyperactivity, hot temper, affective lability, emotional reactivity, disorganization, and impulsivity. Its German version represents one element of the Homburger ADHS Skalen für Erwachsene [44], where it is originally called the Wender Reimherr Interview. The WRAADS has been employed successfully by previous international trials investigating adult ADHD [49].

The Symptom Checklist-90-Standard (SCL-90-S) [50] is a self-report symptom inventory and assesses psychopathology of comorbid conditions and subjective distress. Its design allows for appropriate use in community samples as well as for medical or psychiatric patients and employs nine paramount dimensions (somatization, obsessive-compulsive, interpersonal sensitivity, depression, anxiety, hostility, phobic anxiety, paranoid ideation, psychoticism) and three summary scores (global score, positive symptom distress index, and positive symptom total). The SCL-90-S achieves reliability indices of α = 0.76–0.98 and has been validated well through corresponding diagnostic measures [50].

The Adult ADHD Quality of Life Scale (AAQOL-29) [51] is a 29-item tool developed to assess ADHD-specific quality of life aspects such as life productivity, relationships, life outlook, and psychological health. The AAQOL-29 has been validated using samples from the United States and Europe and has been implemented by several clinical trials concerning adult ADHD (e.g., [52]).

The Childhood Trauma Questionnaire (CTQ) [53] is a five-scale self-report measure, assessing emotional abuse, physical abuse, sexual abuse, emotional disregard, and physical disregard. Research shows significant correlations between childhood trauma and ADHD, using the CTQ to investigate experiences of victimization [54].

The Pittsburgh Sleep Quality Index (PSQI) [55] is a self-assessment questionnaire which surveys sleep quality and disturbance over the course of 1 month; 19 individual items aggregate to seven subscales (subjective sleep quality, sleep latency, sleep duration, habitual sleep efficiency, sleep disturbance, sleeping medication, daytime dysfunction) and yield one global score. This measure is being implemented because research shows associations between adult ADHD and sleep problems [56]. Further, by applying the PSQI, we will be able to examine if the study’s individual treatment has improving effects on symptoms of insomnia in the course of transfer effects.

The State-Trait Anger Expression Inventory 2 (STAXI-2) [57] is an assessment tool to evaluate intensity of state anger, trait anger, anger expression–out, anger expression–in, anger control–out, anger control–in, and an anger expression index. Research shows that ADHD is related to significantly higher levels of state and trait anger [58], which is why we chose to assess anger management within our study. Exhibiting good reliability scores and being well validated through clinical as well as forensic samples, the STAXI-2 is an internationally employed tool and thus the measure of choice in this case [59].

The *Wortschatztest* (WST) [42] is a vocabulary-based IQ-screening method, enabling rapid assessment of verbal intelligence and appraisal of speech comprehension. Consisting of 40 tasks arranged in rows of increasing difficulty, each test task contains one target word and five distractors. Vocabulary-based tests like the WST (e.g. STW for English speaking countries, [60]) have been shown to be useful screening tools, widely used in clinical practice. This instrument was chosen because it is eligible for patients from the age of 16, which is crucial in terms of our objective.

The International Neuropsychiatric Interview Version 6.0 [61] assesses neuropsychiatric symptoms through a short, structured interview following DSM-IV and ICD-10 criteria. With an administration time of approximately 15 min, the International Neuropsychiatric Interview Version 6.0 is the instrument of choice for psychiatric evaluation, as well as outcome tracking in clinical pharmacological trials and epidemiological studies. Worldwide, it is the most used psychiatric structured diagnostic interview, employed by mental health professionals and health organizations in over 100 countries.

An overview of the psychometric measurements is given in Table 2.

**Table 2:** Measures

	T0	T1 Week 1	T2 Week 12/24 ± 14 days	T3 Week 24/36 ± 14 days	T4 Week 36/48 ± 14 days
	Assessment for eligibility	Study visit: Baseline	Study visit: After step 1	Study visit: After step 2	Study visit: Follow-up
Screening and inclusion/exclusion criteria	X				
Informed consent	X				
Drug screening	X^a^	X^a^	^b^	^b^	^b^
Medical history	X				
Pregnancy test (urine)	X	X^a^	^c^	^c^	^c^
IDA-R Interview	X	X	X	X	X
CGI		X	X	X	X
WRAADS		X	X	X	X
IQ (WST)	X				
M.I.N.I.	X				
WFIRS		X	X	X	X
SCL-90-S		X	X	X	X
AAQoL-29		X	X	X	X
CTQ	X				
PSQI		X	X	X	X
STAXI-2		X	X	X	X
QB Test		X	X	X	X
TMS		X	X	X	X
EEG^d^			X	X	
Adherence				X	
List comorbidity^e^	X	X	X	X	X
Socioeconomic status	X	X	X	X	X
Psychopathology and comorbid conditions^e^ in short study visits during step 1	Week 4 and 8; for waiting list group additionally in week 16 and 20				
Psychopathology and comorbid conditions^e^ in short study visits during step 2	Week 16 and 20; for waiting list group additionally in week 28 and 32				
Safety assessments^e^ during step 2 for methylphenidate treatment	Week 13, 14, 15, 16, 18, 20 and 22, while waiting list group will undergo these safety assessments three months later (week 25, 26, 27, 28, 30, 32 and 34)				
Adverse events according psychiatric adverse events (PAEs)	Weekly/biweekly between T2 and T3				

^a^ According to clinical routine and center standards

^b^Only if drug abuse is suspected

^c^Only if pregnancy is suspected

^d^Only for patients in the NF groups

^e^A study-specific investigator report, which allows the assessment of neurological as well as psychiatric symptoms

*AAQOL-29* Adult ADHD Quality of Life Scale, *CGI* Clinical Global Impression Scale, *CTQ* Childhood Trauma Questionnaire, *EEG* electroencephalogram, *IDA-R* Integrated Diagnostic Scale of adult ADHD – Revised, *M.I.N.I.* 6.0 The International Neuropsychiatric Interview (Version 6), *PSQI* Pittsburgh Sleep Quality Index, *QB* Quantified Behavior Test, *SCL-90-S* Symptom Checklist-90-Standard, *STAXI-2* State-Trait Anger Expression Inventory 2, *T* test time, *TMS* transcranial magnetic stimulation, *WFIRS* Weiss Functional Impairment Rating Scale, *WRAADS* Wender Reimherr Adult Attention Deficit Disorder Scale, *WST* Wortschatztest (vocabulary-based IQ screening)

#### Psychophysiological data

Using EEG and magnet resonance imaging in the subproject ESCAbrain, we aim to obtain potential biological markers and predictors of ADHD to uncover physical effects of possible improvements through treatment. Those assessments will not be administered to all patients, but only to those subgroups receiving the CBT-based counseling program and NF or MPH and NF. Patients will undergo those procedures before they enter stage 2 of the program and after they have been randomly assigned to subsequent treatment groups.

Using EEG measures, we expect to find predictors of ADHD within the frequency profile (spontaneous theta band and alpha band activity) and the intensity of preparatory cognitive activity (CNV amplitude). As suggested by relevant literature, said indicators represent promising biological markers of ADHD and explain about 30% of variability in behavioral improvement following NF treatment [62]. The magnetic resonance imaging-based predictors that we hope to identify are part of the fronto-stratial connection (fractional anisotropy) and volumetric grey matter density of dorsolateral-prefrontal and striatal regions that have previously been associated with ADHD (e.g., [63]).

To uncover the underlying mechanisms constituting improvement through NF training or counseling and pharmacological treatment, EEG recordings will be repeatedly conducted following the second step treatment phase. Changes in electric brain activity (hypothesized reduction of resting theta activity and increased CNV activity) will be treated as additional secondary outcome measures for the respective subgroups.

All participants and parents/guardians will be informed about the possibility of those additional assessments in advance through general patient information. Patients assigned to one of the affected groups will additionally be provided with a separate patient information sheet and asked to give informed consent.

At three points in time during our investigation (T1, T2, T3), we intend to implement transcranial magnetic stimulation. This method permits a very accurate record of brain activity, aiming to elucidate processes that could contribute to problems associated with ADHD. Transcranial magnetic stimulation will be applied using a Medtronic MagPro X100 Stimulator with MagOption (Medtronic, Denmark). A figure-of-eight coil with a diameter of 65 mm will be placed onto the patient’s head above the supposed hand area of the motor cortex. Surface electromyography will be recorded from the contralateral FDI with a standard electromyographic amplifier (Medtronic Keypoint 4; recording software: Medtronic Keypoint V 5.01). Filters will be set to a bandwidth of 1 Hz and 10 kHz, respectively. The optimal position of the coil will be determined through moving the coil by 0.5 cm steps until an optimal Motor Evoked Potentials (MEP) can be registered. Resting motor threshold (RMT) and active motor thresholds will be determined according to protocol, as suggested by Kujirai et al. [64].

Further, the Quantified Behavior Test [65] will be employed. This tool assesses cognitive and behavior domains (attention and impulsivity) of adult ADHD through a continuous performance task, while simultaneously recording motor activity using infrared motion tracking techniques.

### Methods against bias

In order to guarantee blinded treatment allocation, an independent research center, not otherwise engaged in this investigation, will carry out randomization (Clinical Trials Unit, University Medical Center Freiburg). Applying equal allocation ratios to all three conditions at step 1 (step 2), the process will be stratified by the center for both steps and by partial/non-response criteria in step 2. Statistical analyses will be conducted using the conservative intent-to-treat population in order to avoid statistical bias. Corresponding evaluation plans will be determined prior to analysis. Primary (IDA-R) and secondary outcomes will be assessed by investigators who are blind to the patient’s study status. To ensure reliability of those assessments, investigators will receive appropriate training prior to study initiation. In addition, supervised psychosocial treatments are being inaugurated before launching the study. During PE, two sessions will be videotaped and evaluated by an independent rater. Using a structured instrument, the content of each session of PE as well as counseling will be documented.

### Data management

The Clinical Trials Unit of the University Medical Center Freiburg will provide electronic questionnaires within the framework of a remote data entry system (RDE-LIGHT), while also administering the database. RDE-LIGHT is a proprietary remote data entry system based on HTML forms, which is developed, validated, and maintained by the Clinical Trials Unit of the University Medical Center Freiburg. Technical specifications of the trial database, such as variable names and formats, will be documented in a database manual. Every study center is asked to carry out the documentation in the electronic case report form as soon as possible. A user manual for the RDE-LIGHT system will be provided as this will be individualized for each study within the ESCALife trial. Every investigator will be trained in using the documentation system. The data will be reviewed for completion, consistency, and plausibility using the Statistical Analysis System (SAS^©^) software.

### Central randomization

Patients fulfilling the eligibility criteria will be randomized by the Randomization and Data Management Office of the Clinical Trials Unit of the Medical Center – University of Freiburg. The randomization procedure has been described in above. The randomization code will be produced by validated programs based on the SAS^©^.

### Analysis

#### Proposed sample size and power calculation

Based on available evidence, sample size was calculated in order to allow confirmatory evaluation of the effect of PE versus control (waiting list) at step 1 with a presumed effect size of 0.45 (change in IDA-R score from T1 to T2 after intervention/waiting, software: STPLAN Version 4.3). Conducting a two-sided *t* test aiming to reach a power of 80% and a significance level of 5%, per group, 79 patients are required to fully pass through study protocol in order to detect a difference when the true effect size is d = 0.45. With a 1:1:1 allocation ratio for step 1, this yields a power of 24% to detect a difference between TASH and control when the true effect size is d = 0.2 (exploratory evaluation of pilot data). To account for the possibility that some patients (15%) will provide incomplete data at T2, a total of 279 patients should be randomized at step 1. We estimate that approximately 400 patients will have to be screened in order to identify enough patients suitable for study participation.

Concerning drop-out rates, previous pharmacological trials with observation periods up to 6 months showed that premature termination of attendance occurred for 15% of patients within the first 3 months. Another 15% dropped out within the following 3 months [45, 66]. Therefore, 190 patients will be randomized to the pilot investigations of NF in step 2, about 47 for each intervention. Based on a conservative estimate of 85% of patients providing complete data at T3, this yields a power of 34% to detect an effect of the addition of NF as compared to no NF in partial and non-responders, respectively, when the true effect size is d = 0.3.

Participants will be recruited via outpatient ADHD centers adjoined to university hospitals and the respective research institutes involved in the umbrella project ESCAlife. Patients participating in our routine diagnostic and treatment program will be screened for eligibility and recruited accordingly. The average patient flow is estimated to be between 150 and 300 new ADHD patients per year and center. Thus, we should be able to acquire as many patients as needed (*N* = 279) to achieve appropriate statistical power. To reach that goal, each center will need to recruit 25 patients per year, which seems reasonable and feasible, especially considering the expertise and experience of the centers involved. For instance, the centers of Freiburg (now Oldenburg, Prof. A. Philipsen), Würzburg and Homburg/Saar conducted a multisite ADHD study funded by BMBF (*Bundesministerium für Bildung und Forschung*, Registration: CCT-ISTRCTN54096201 and CCT-ISRCTN73911400). The majority of centers engaged in this study were also involved in large pharmacological trials, e.g., the EMMA study, with a sample size of *N* = 359 [45].

An overview of the recruitment numbers is given in Table 3.

**Table 3:** Patient distribution

	Homburg	Mainz	Oldenburg	Tübingen	Rostock	Mannheim
Number of newly admitted outpatients with ADHD aged 16 years or above	*n* = 200	*n* = 160	*n* = 210	*n* = 200	*n* = 150	*n* = 150
To be assessed for eligibility in the 2-year recruitment period (*N* = 400)	*n* = 100	*n* = 80	*n* = 120	*n* = 100	*n* = 90	*n* = 90
To be recruited (*N* = 300)	*n* = 75	*n* = 60	*n* = 80	*n* = 75	*n* = 60	*n* = 60
To be randomized (*N* = 279)	*n* = 50	*n* = 40	*n* = 50	*n* = 50	*n* = 50	*n* = 40

#### Primary analysis and baseline data

Primary analysis will be conducted according to the intention-to-treat principle [67]. This means that data provided by patients will be analyzed according to the treatment condition to which they were allocated, irrespective of whether they refused or discontinued treatment or whether other protocol violations were revealed.

The per-protocol (PP) population is a subset of the full analysis set, defined as the group of patients who had no major protocol violations, received a predefined minimum dose of treatment and underwent examinations required for outcome assessment at relevant, predefined times. To be included in the PP population, the patients would have to participate in at least six of eight PE or TASH sessions, as well as 20 of 25 NF sessions. Sensitivity analysis of said PP population will be performed [67].

Before including the first patient, a detailed statistical analysis plan will be prepared and, at the latest, completed by the time the data is (blindly) reviewed. If the statistical analysis plan contains any changes to the analyses outlined in the trial protocol, they will be marked as such and reasons for amendments will be given.

All statistical programming for analysis will be performed by the SAS®.

Demographic and other baseline characteristics will be summarized descriptively for all patients.

Continuous data will be summarized by arithmetic mean, standard deviation, minimum, 25% quantile, median, 75% quantile, maximum, and the number of complete and missing observations. If deemed appropriate, continuous variables can also be aggregated into categories.

Categorical data will be summarized with respect to the total number of patients in each category and the number of missing values. Relative frequencies are displayed as valid percentage (number of patients divided by the number of patients with non-missing values).

#### Primary outcomes

Primary statistical analyses of steps 1 and 2 will be conducted by intention to treat, so that all randomized patients will be analyzed according to their allocated groups. Changes in the IDA-R score between T1–baseline and T2 (after interventions/waiting) or T2–baseline (before randomization to ± NF) and T3, respectively, will be evaluated in separate mixed-effects models for repeated measures (MMRM [68]). The MMRMs will include fixed categorical effects of treatment, center, visit, and treatment-by-visit interaction as well as continuous and fixed covariates of baseline and baseline-by-visit interaction. Further, covariates, which are predictive of missing values, will be included based on a pre-specified selection strategy to correct for potential bias arising from missing data. Unstructured covariance matrices will be used to model within-patient correlations (a prospective alternative strategy will be devised to address potential convergence problems). Comparison of primary treatment change scores at T2 and T3 will be based on least-squares means with two-sided 95% confidence intervals. Evaluation of PE will be conducted through confirmatory factor analysis. Subgroup analyses will be conducted in an exploratory manner by including interaction terms in the MMRMs, focusing on the analysis of psychosocial and neurobiological predictors. Particularly, sex effects will be investigated as prognostic and predictive factors.

Potential biological predictors are initially assessed separately for each predictor and treatment through examination of their correlation with clinical improvement as well as planned comparisons, aiming to identify age- and treatment-specific predictor patterns. Next, multivariate pattern classification will be applied, combining markers from different modalities in order to optimize prediction.

#### Secondary outcomes

Secondary outcomes will be analyzed descriptively in a similar fashion as the primary outcome, using regression models as it is appropriate for the respective type of data. No confirmatory analysis of any of the secondary outcomes is planned. Treatment effects will be calculated employing two-sided 95% confidence intervals. Details will be specified in the Statistical Analysis Plan, which will be prepared before the inclusion of the first patient.

For all outcome scores (IDA-R, WRAADS, WFIRS-S, AAQoL-29, PSQI, SCL-90-S, STAXI-2), for changes from T1 to T2 (intervention groups: after interventions, control group: after waiting, before TASH) at step 1, and for changes from T2 (before step 2) to T3 (after interventions) at step 2, an analysis will be conducted similarly to the primary outcome analysis for continuous measures. Follow-up assessments of full responders after step 1 will be evaluated descriptively.

Scores will be calculated according to the respective manual.

The relationship between childhood trauma (measured by CTQ) and the severity of ADHD at baseline (defined by IDA-R) will be analyzed using Spearman correlation coefficients and linear regression.

Likewise, the relationship between anger management (measured by STAXI-2) and the severity of ADHD at baseline (defined by IDA-R) will be analyzed using Spearman correlation coefficients and linear regression. First, it will be assessed whether anger management is influenced by allocated therapies (similar to primary outcome analysis). The relationship between anger management at baseline (as measured by STAXI-2) and the efficiency of the respective treatment, measured by the difference in severity of ADHD (as defined by IDA-R) from T0 and T1 and from T2 and T3, will be analyzed using linear regression.

#### Missing values

As few patients as possible should discontinue treatment, while all patients who drop out should be followed up regardless in order to record data as required by the intention-to-treat principle.

Mean changes from baseline will be analyzed using a restricted maximum likelihood-based repeated measures approach. Details will be specified in the Statistical Analysis Plan.

### Quality assurance and monitoring

Monitoring is being performed by the Clinical Trials Unit of the University Medical Center in Freiburg. Adapted monitoring will be done according to ICH-GCP E6 and standard operating procedures. All monitoring procedures, such as frequency of visits, and source data verification will be predefined in the monitoring manual. All information recorded on case report forms must be traceable to source documents in the patient’s file; the original documents will be kept.

### Legal foundation and inclusion of the ethical committee

The study will be conducted in accordance with the ICH-GCP guidelines and the Declaration of Helsinki. The study was approved by the local ethic committee. All substantial changes in the trial protocol or complications and severe adverse events will be reported to the ethics committee.

### Stopping rules

Key stopping rules for patients are a withdrawal of informed consent or unwillingness to further participate in the trial or any factors affecting the patient’s well-being. Key stopping rules for the trial will be if data suggests a revision of the risk-benefit ratio.

## Discussion

Results of our current trial will underpin the recommendations as presented by the evidence-based and expert-derived treatment guidelines (NICE 2009) with empirical data. Thus far, evidence for non-pharmacological treatment options in adults with ADHD is limited. From the view of primary healthcare, predicting ADHD patients’ individual response to relatively low-level psychosocial interventions (e.g., PE, TASH) or to NF, which are widely used treatment options in clinical practice, is a prerequisite for individualized efficacious therapy in ADHD. In this regard, we expect that PE and TASH may have beneficial effects in terms of the adult-specific ADHD psychopathology and related symptom domains, e.g., emotional dysregulation and/or disorganization. If differences between PE and TASH treatment are found, we expect them to be very small and thus negligible. Moreover, our study aims to contribute to current research regarding the potential of NF as a treatment for adults and late adolescents with ADHD.

There are two aspects to be investigated through our stepped care program. First, the main question is whether NF in combination with counselling will display larger effects than counselling alone. Effects between *d* = 0.3 and 0.5 are to be expected. The second aspect investigated, refers to the effects of NF in comparison to the well-known effects of MPH treatment. In light of the different modes of action in both treatments we hypothesize that NF and MPH might have additive effects and thus a combination will yield bigger effects as compared to MPH alone.

Another aim of the study is to identify patients who are in need of pharmacological treatment. In order to do so, we re-evaluate our patients after they complete step 1 with respect to therapeutic response and remaining ADHD psychopathology as measured by the IDA-R. If a patient scores 18 or lower on the IDA-R, they will be classified as full responders. An average item score of this 18-item rating scale of ≤ 1 indicates subclinical presence of ADHD. Non-response is given if the total score of the IDA-R is ≥ 28, corresponding to an average item score of ≥ 1.5. With reference to our population norms, which have been acquired within the last decade, a score of 28 has a *t* value of 81, which is more than two standard deviations from standard population mean. Partial responders are defined as patients scoring 19–27 on the IDA-R, reflecting mild to moderate ADHD. Different interventions are assigned to full responders, partial responders, and non-responders. We hypothesize that symptom severity may be a predictor for the appropriateness of different treatment options.

We aim to study different psychosocial and neurobiological predictors of therapeutic response or non-response. Possible sex effects and the influence of biological rhythms like sleep patterns are of specific interest in this context. Additionally, childhood adversity, social influences, and typical comorbid conditions will be analyzed regarding treatment outcome at several steps of treatment. These factors are relevant aspects influencing the pathogenesis of ADHD but have not yet been investigated within RCTs concerning late adolescents and adults.

Data regarding the effects of different interventions at different stages of treatment as well as the identification of possible predictors may open the possibility to individualize treatment concepts. This may help to implement treatment decisions more effectively and lower healthcare expenses. Further, identification of optimal, individualized treatment combinations and concomitant improvement in terms of psychopathology and functional status will obviously benefit the individual patient.

Another objective of this investigation is the identification of typical problems arising when transitioning from adolescence into adulthood for patients with ADHD. Thus far, neither adult patients nor adolescents have been included in treatment studies in order to evaluate efficacy and tolerability of pharmacological or non-pharmacological approaches. The age criterion for patient inclusion in the proposed study is 16 to 45 years, which closes the gap between adolescence and adulthood. Therefore, we have the opportunity to compare late adolescents with young adults and to study the effects of different treatments over a life span that is crucial for personal and social development.

### Trial status

Protocol version 01 dated 8 July 2015. Latest protocol version 05 dated 20 December 2016. Date of recruitment start: 28 January 2016. Date of recruitment end: 31 October 2019.

## Additional file

Additional file 1: Spirit 2013 Checklists. (DOC 121 kb)

## Abbreviations

AAQOL-29: Adult ADHD Quality of Life Scale
ADHD: attention-deficit/hyperactivity disorder
CBT: cognitive behavioral therapy
CGI: Clinical Global Impression Scale
CNV: contingent negative variation
CTQ: Childhood Trauma Questionnaire
EEG: electroencephalogram
ESCA: Evidence-Based, Stepped-Care of ADHD
ESCAlate: Evidence-Based, Stepped-Care in Late Adolescents and Young Adults with Attention-Deficit/Hyperactivity-Disorder
ESCAlife: Evidence-Based, Stepped-Care of ADHD along the life-span
IDA-R: Integrated Diagnostic Scale of adult ADHD – Revised
MMRM: mixed-effects models for repeated measures
MPH: methylphenidat
NF: neurofeedback
PE: psychoeducation
PP: per protocol
PSQI: Pittsburgh Sleep Quality Index
RCT: Randomized Controlled Trial
RDE-LIGHT: Remote Data Entry System provided by Clinical Trials Unit Freiburg
SAS©: Statistical Analysis System
SCL-90-S: Symptom Checklist-90-Standard
SCPs: slow cortical potentials
STAXI-2: State-Trait-Anger-Expression-Inventory-2
T1: test time 1
T2: test time 2
T3: test time 3
T4: test time 4
TASH: telephone-assisted self-help
WFIRS: Weiss Functional Impairment Rating Scale
WRAADS: Wender Reimherr Adult Attention Deficit Disorder Scale
WST: *Wortschatztest* (vocabulary-based IQ screening)
